# Testing a novel method for improving wayfinding by means of a P3b Virtual Reality Visual Paradigm in normal aging

**DOI:** 10.1186/s40064-016-2978-7

**Published:** 2016-08-09

**Authors:** Marina de Tommaso, Katia Ricci, Marianna Delussi, Anna Montemurno, Eleonora Vecchio, Antonio Brunetti, Vitoantonio Bevilacqua

**Affiliations:** 1 Applied Neurophysiology and Pain Unit, SMBNOS Department, Policlinico General Hospital, Bari Aldo Moro University, Giovanni XXIII Building, Via Amendola 207 A, Bari, Italy; 2 Department of Electrical and Information Engineering, Polytechnic of Bari, Bari, Italy

**Keywords:** Virtual reality, Event-related potentials, Virtual wayfinding, Aging

## Abstract

**Background:**

We propose a virtual reality (VR) model, reproducing a house environment, where color modification of target places, obtainable by home automation in a real ambient, was tested by means of a P3b paradigm. The target place (bathroom door) was designed to be recognized during a virtual wayfinding in a realistic reproduction of a house environment. Different color and luminous conditions, easily obtained in the real ambient from a remote home automation control, were applied to the target and standard places, all the doors being illuminated in white (W), and only target doors colored with a green (G) or red (R) spotlight. Three different Virtual Environments (VE) were depicted, as the bathroom was designed in the aisle (A), living room (L) and bedroom (B). EEG was recorded from 57 scalp electrodes in 10 healthy subjects in the 60–80 year age range (O—old group) and 12 normal cases in the 20–30 year age range (Y—young group).

**Results:**

In Young group, all the target stimuli determined a significant increase in P3b amplitude on the parietal, occipital and central electrodes compared to frequent stimuli condition, whatever was the color of the target door, while in elderly group the P3b obtained by the green and red colors was significantly different from the frequent stimulus, on the parietal, occipital, and central derivations, while the White stimulus did not evoke a significantly larger P3b with respect to frequent stimulus.

**Discussion:**

The modulation of P3b amplitude, obtained by color and luminance change of target place, suggests that cortical resources, able to compensate the age-related progressive loss of cognitive performance, need to be facilitated even in normal elderly. The event-related responses obtained by virtual reality may be a reliable method to test the environmental feasibility to age-related cognitive changes.

## Background

The progressive extension of human survival in occidental society generates new problems in the organization of daily living activities and optimal environmental context, where the residual capabilities may be facilitated and even promoted in the presence of cognitive dysfunction.

In normal aging, visual recognition of daily life stimuli is impaired with respect to young people, depending upon the intrinsic characteristics of the object and the context in which this is embedded (Mateus et al. 2013; Rémy et al. 2013; Mott et al. 2014). Special recognition is a fundamental element to achieve a good orientation in different environments, like a house, hospitals, and health facilities, in order to facilitate activities in daily living. Spatial disorientation and reducing wayfinding abilities is early and invalidating symptoms of dementia. Architectural wayfinding for old people and dementia were also studied with regard to nursing home, where structural design, as well as furniture, light, and colors, may be adapted to dementia—friendly environment model (Marquardt 2011). However, the prevention of progressive loss of functional capability may be reached through the adaptation of his environment to the sensory and cognitive dysfunctions, caused by aging and dementia, in order to prolong the permanence at home and avoid institutionalization. Moreover, wayfinding would be improved also in hospitals, where aged people are often disoriented and prone to incongruent behaviors. Some easy modifications of ambient color and luminance may be obtained by home automation, which may improve the wayfinding enabling the remote control of places lights and luminance (Denti 2014). In order to design the best conditions to be applied for places recognition improvement, Virtual Reality (VR) techniques may efficaciously reproduce home, hospital or nursing house ambient, enabling the extraction of cognitive response to environment features changes. In fact, there are recent evidence on the reliability of cognitive-related responses obtained by oddball paradigms realized in VR frames (Chapman and Bragdon 1964; Sutton 1965; Bayliss 2003; Polich 2007), which seems to be a solid method to explore the capacity to recognize a target place during a virtual wayfinding (Bayliss 2003). In theory, VR might test the real environment’s impact on cognitive capacities in aging and dementia, as well as any change easily produced by home automation control to improve wayfinding even in unknown places. So far, the main aims of the present study are

1. The validation of a model of VR consisting of the reproduction of a real house environment modified by home automation as a reliable stimulus to obtain a P3b response, similar to that resulted from standard visual oddball paradigm (Polich 2007)
2. The preliminary testing of this novel model in a cohort of young and old healthy subjects, in order to prove possible age-related changes of target places recognition in response to ambient condition changes obtained by home automation techniques. This in view of a possible application in mild cognitive impairment and dementia to make easy and rapid changes of home or hospital environment for a better cognitive impact and facilitation of daily living activity.

## Methods

After the visual and automatic inspection of EEG tracks, four cases in the O and 2 cases in the Y groups were discarded because of the low quality of the recordings; so the final evaluation was carried out in 4 F and 6 M in the O group (mean age 65.4 ± 8.23) and 5 F and 7 M in the Y group (mean age 25 ± 3.8).

### Virtual reality method: home environment reconstruction

The requalification started with the acquisition of the real environmental parameters, such as room dimension and their shape. The starting real environment was a laboratory of the Polytechnic of Bari. The rooms, after they were modeled in Virtual Reality, were furnished as a living environment and illuminated with different spot lights, by means of domestic remote control. The steps needed to create a virtual model were:

- Acquisition of real environmental measurements through a high precision laser (Leica 3D Disto);
- The points acquired by the laser meter were exported to a CAD file, processed using Autodesk Inventor, from which a rough 3D model of the environment was created;
- The rough model was enhanced with furniture, so the model was made look like a home environment containing two corridors, three rooms (living room, bedroom, and a kitchen) and a bathroom, positioned in the corridors.

**Fig. 1:**
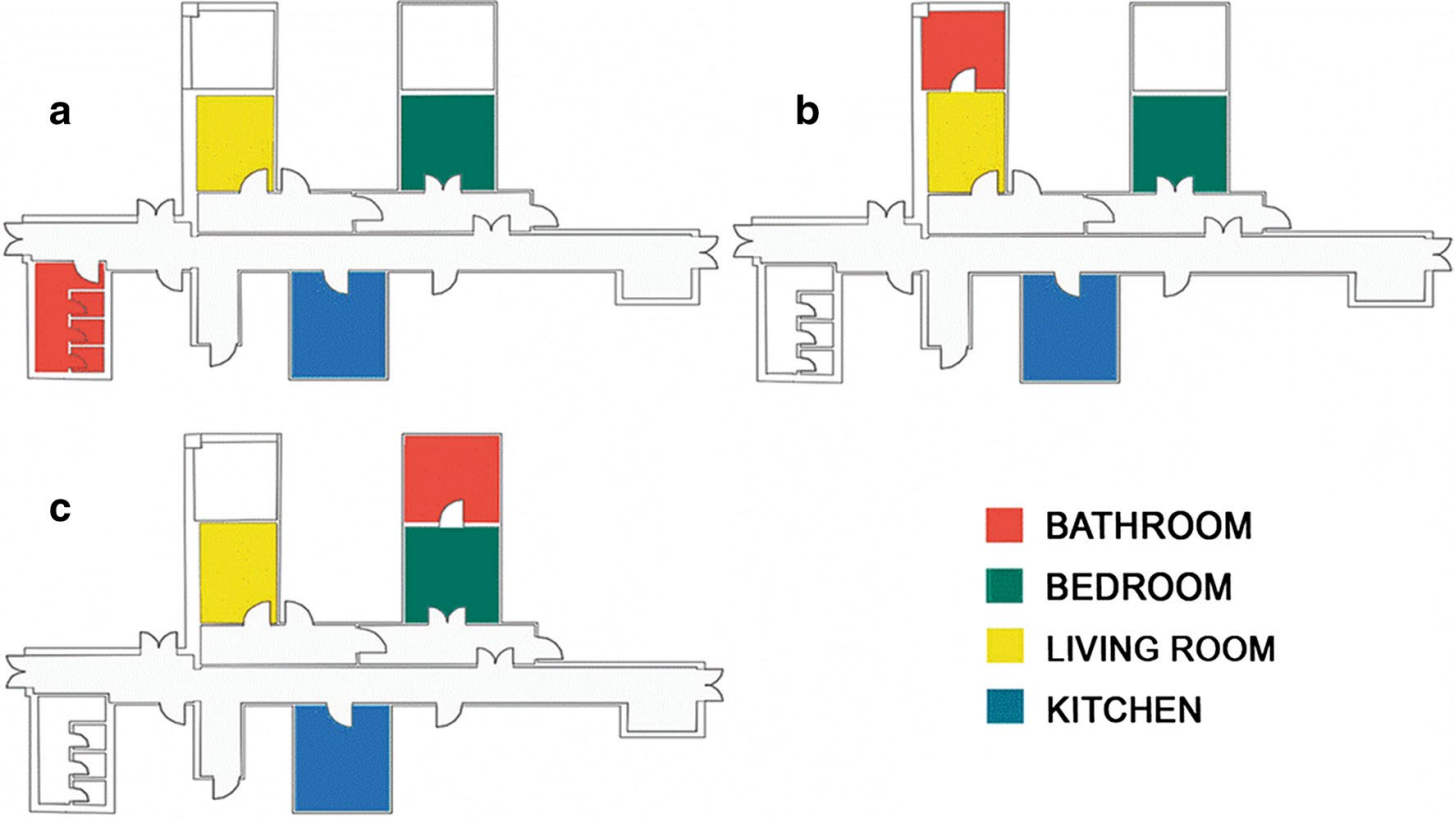
The design of virtual environment is reported. **a** Bathroom door in the corridor. **b** Bathroom door in the living room. **c** Bathroom door in the bedroom

**Fig. 2:**
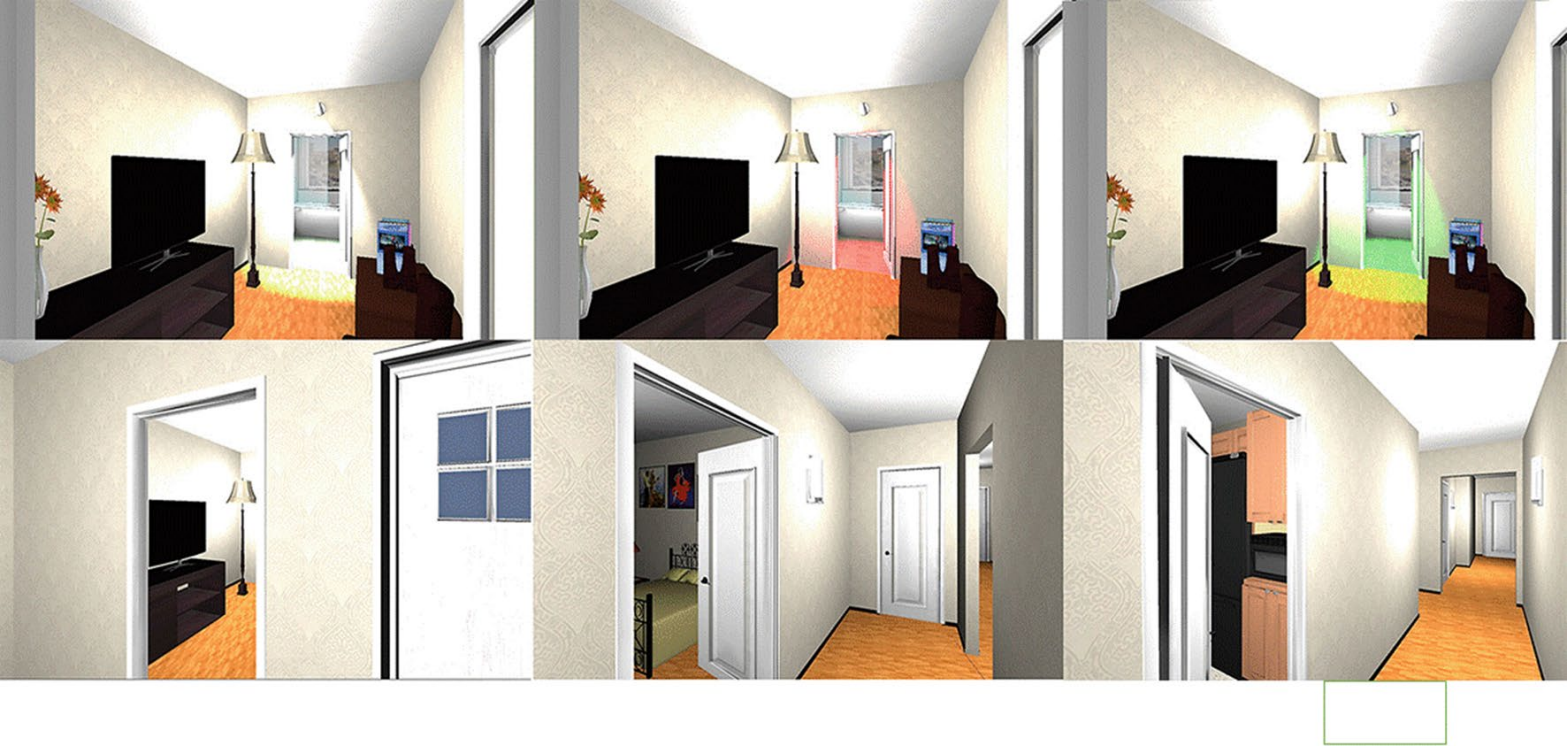
Examples of virtual scenarios during the simulated walking. At the top, the different light spot is reported

From the basic model (Fig. 1a), other two different models were created, where each one contained the bathroom,—the target place—in a different position, such as in the living room (Fig. 1b), and in the bedroom (Fig. 1c). Starting from each object, three different scenes were generated, each containing one of the three models mentioned above (Fig. 2).

### Visual stimulation

**Fig. 3:**
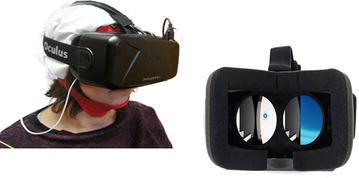
The model of ocular rift is reported

In order to immerse the user in the VR scenes, an Oculus Rift DK2 was used, which is a virtual reality head-mounted display headset developed by Oculus VR. For the purpose of this project, when the user wore the device, he saw the virtual scene through which the visual stimulation occurred. The binocular vision describes the way in which we see two views of the world simultaneously; the view from each eye is slightly different and our brain combines them into a single three-dimensional stereoscopic image, an experience known as stereopsis. The Oculus Rift presents two images, one for each eye, generated by two virtual cameras separated by a short distance (Fig. 3).

The Oculus Rift DK2 employs an OLED panel for each eye, with a resolution of 960 × 1080 and a refresh rate of 75 Hz (it globally refreshes, rather than scanning out in lines). The panels have low persistence, meaning that they only display an image for 2 ms of each frame. This combination of higher refresh rate, global refresh, and low persistence means that the user experiences none of the motion blurring or judders that are experienced on a regular monitor. It uses high-quality lenses to allow for a wide field of view. The separation of the lenses is adjustable by a dial on the bottom of the device, in order to accommodate a wide range of interpupillary distances. In order to work, the Rift was connected by a cable to a PC equipped with a powerful GPU, at least, equivalent to an NVIDIA GeForce GTX 970 or AMD R9 290, and a CPU, at least, equivalent to Intel i5-4590.

### EEG recording

In addition to the 19 standard positions of the international 10–20 system, 38 additional electrodes were placed on the x, y, and z coordinates provided by the Advanced Source Analysis (ASA) software (ASA version 4.8.1; ANT Software, Enschede, The Netherlands;httpHYPERLINK “http://www.ant-neuro.com/”://www.ant-neuro.com; http://www.ant-neuro.com). The reference electrode was placed on the linked mastoids, the ground electrode was in Fpz and two electrodes were placed above and below the right eyebrow for electrooculogram (EOG) recording. The impedance was kept at 5 kms or less. The EEG and EOG signals were amplified with a bandpass filter of 0.5–80 Hz digitized at 250 Hz and stored on a biopotential analyzer (Micromed System Plus; Micromed, Mogliano Veneto, Italy; http://www.micromed-it.com).

### Experimental procedure

During the sessions, the participants sat on a reclining lounge chair located in a sound-attenuated, electrically shielded and dimly lit room. At the start of the sessions, they were asked to avoid blinking as much as possible. The study was approved by the Ethic Committee of Bari Policlinico General Hospital. All subjects were informed about the procedure and signed an informed consent. The trial was included into the Rescap Apulian Living Lab project (http://livinglabs.regione.puglia.it/).

### P3b paradigm

The P3b paradigm was designed in order to test the best features of a target place to be recognized in a virtual ambient reproducing a real house. We selected the bathroom door as target stimulus, for its presence in all houses and health facilities, where an elderly subject could have difficulty in moving toward this room essential for daily living abilities. In the virtual environment, that simulates a home, all rooms that were different from the bathroom were identified as frequent stimuli (F) while the rare stimulus was assigned to the bathroom room itself. To differentiate the target stimulus from the frequent ones, and to understand the better features for target room doors recognition, bathroom doors were semi-opened, in order to allow the recognition of the typical furniture. In addition, different color and luminous conditions, easily obtained in the real ambient from a remote home automation control, were applied. Bathroom doors were illuminated in white, like the other non-target doors (W), or colored with a green (G) or red spotlight (Fig. 2). Subjects were informed that they would perform a virtual walking through an apartment, looking for the bathroom door, and were requested to press a button as soon as the bathroom door appeared in the field of view. To make the experiment easier, navigation inside the environment was automatically controlled and with a fixed duration; in this way, rare and frequent stimuli were proposed in succession and in a controlled number. In order to examine the best bathroom position for being recognized within the virtual house, three different Virtual Environments (VE) were depicted, as the bathroom was designed in the aisle (A), living room (L) and bedroom (B). Each VE constitutes a single block (Fig. 1), where each block included 30 rare stimuli (10 for each color spotlight) and 120 frequent ones (all other rooms doors). Thanks to the anchors inserted in VE, the system computed random virtual paths that, alternatively, started from the bathroom, end up to another place of the house and vice versa, in order to have a rare stimulus followed by a certain number of frequent ones.

During the automated navigation in VE, whenever an open door entered the field of view of the virtual camera, a trigger was launched to the EEG recording system according to the door type; in particular, bathroom door throws rare trigger, with different values depending on the spotlight colors, while the doors of the remaining three rooms launch the same frequent trigger value.

**Fig. 4:**
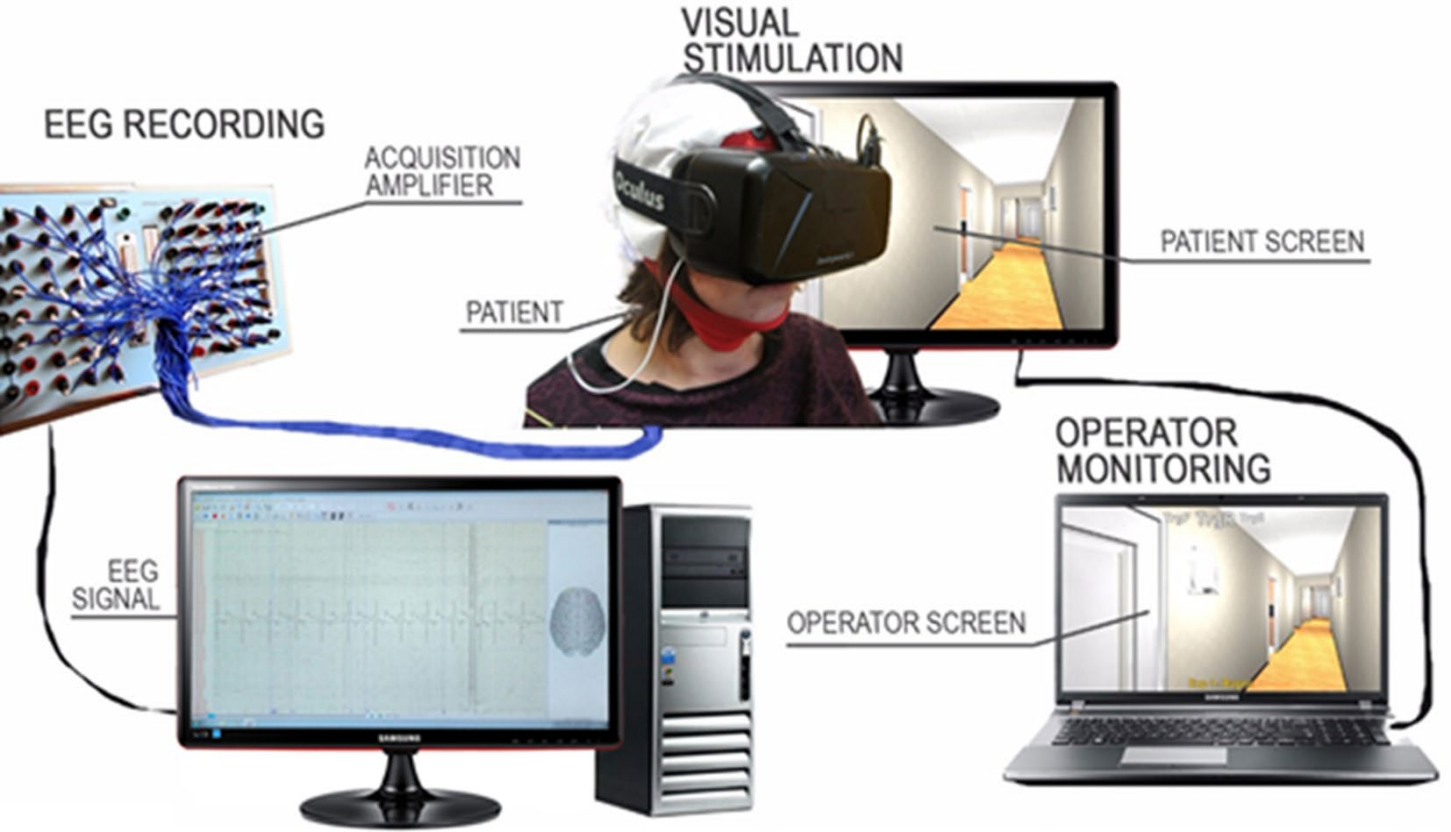
The stimulation apparatus, connected with the EEG amplifiers

During the experiment, a virtual agent, equipped with a virtual camera that reproduces its visualization on the user’s head-mounted display, followed the route. In order to allow the health technicians to supervise the experiment evolution, an operating mode of the application was developed. In this modality, the operator saw the same virtual environment visualization of the patient and other information related to acquisition protocol. A monitor, used exclusively by the operator, was in fact employed. In operator display, trigger information showed which type of stimulus was visible in the field of view and, accordingly, which trigger code was sent to EEG recording system. Other information provided to the operator were the number of stimuli sequence block, and the next destination, e.g. “bathroom” and “other place” (Fig. 4).

We have also to outline that the color modifications reproduced in VR were realized in the real ambient, by means of different lights activated by a remote control.

In order to test the reliability of the P3b obtained in the virtual ambient, subjects were also submitted to a standard oddball visual paradigm presented on a monitor in a fixed position, with the same percent rate of the target and no target stimuli. This recording was performed before or after the VR paradigm, in a randomized inter-subject way.

### Data analysis

We measured the positive component (P3b) to detect the neural activity related to the target stimulus. Averaged epochs were 1100 ms, beginning 100 ms before stimulus onset. Waveforms were averaged off-line after visual inspection, such that trials in which the EEG or EOG exceeded ±65 uV were rejected by the automated procedure provided for artifacts by the ASA-ANT software. In addition, the Independent Component Analysis (ICA), provided by ASA software, was applied to further subtract from EEG tracks the features of the visually inspected EEG artifacts and EOG. We evaluated P3b for each presented target stimuli color (W, R, and G) in each block (A, L, B). We averaged responses to W, R, G and F stimuli in the three virtual environments (A, L, and B). Each averaged response was visually inspected and compared to the responses obtained in the fixed-display condition. Only subjects with at least 7 artifacts free responses for each presented target stimuli color in each block, who in addition did not show substantial modification of P3b latency and amplitude with respect to the standard visual oddball paradigm, evaluated by visual inspection, were admitted to the final analysis. Absolute latencies and amplitudes were automatically computed by ASA software at the highest positive peak in the time range 300–600 ms after stimuli delivering. The amplitude of each wave was measured from the baseline. A grand average (GA) of ERP was performed in the two groups:

1. Fourteen subjects 7 M and 7 F in the 60–80 years age range (O—old group).
2. Fourteen subjects 7 M 7 F in the 20–30 years age range (Y—young group).

All subjects were selected from hospital and University staff or were familiars of these. Exclusion criteria were the absence of history or objective signs of any general medical, neurological and psychiatric disorders, including a Mini Mental State Evaluation Score (Folstein et al. 1975) <26 for subjects in the old group, and central nervous system acting drugs taking. The inclusion criterion was a scholar age of at least 13 years. Patients under treatment with mild hypertension were admitted. The Mini-Mental test score in older patients was 27.32 ± 1.27, with a mean secular age for older patients of 14.57 ± 2.20 and for young participants 15.14 ± 2.07 years (ANOVA F 0.44, n.s.). Visual acuity was also measured by an oculist, and only subjects with normal visual function in natural conditions are obtained by contact lens or glasses (which were allowed during the task) were admitted.

### Statistical analysis

In order to evaluate the changes of P3b amplitude with regard to the color of the target stimulus, the different virtual environment (aim 1) and the two age groups (aim 2), we employed MANOVA test. In the first step analysis, we introduced the amplitudes of the artifact-free maximal positivity in the time range 300–600 music recorded over the employed 57 EEG derivations as variables in the MANOVA test, where groups Young versus Old and target stimuli W versus R versus G v’s F were the main factors, also evaluating the interaction between groups × target stimuli. In a second step analysis, we introduced the same variables as in the first step, considering the effect of Virtual Environments (A vs. L vs. B) on the P3b by target stimuli, and the interaction VE × groups. The P3b latencies were evaluated by one-way ANOVA, considering Pz derivation as variable and the group as a factor. A posthoc Bonferroni test was run out in single groups to test significant differences of P3b amplitudes and latencies among target stimuli and Virtual Environments. Scalp Maps reporting the Grand Average of the P3b in single groups, and Statistical Probability Maps (SPM) reporting the results of Bonferroni test, were constructed according to the tri-dimensional scalp model provided by ASA software.

## Results

A reliable P3b response was obtained in 22 cases, as reported above. In single subjects, the amplitudes and latency values of the P3b obtained in VR condition did not exceed a 10 % difference from that obtained in the fixed position situation by the standard visual oddball paradigm.

### P3b Latency

P3b latency was not significantly changed as an effect of the group and the different visual stimulations (Table 1).

**Table 1:** P300 latencies on Pz derivation in old (Ag) and young (Y) groups, in aisle (A), living room (L) and bedroom (B) virtual environment (VE)

VE stimulus	Mean (ms)	SD
AG		
A		
White	430.04	30.32
Red	411.46	33.52
Green	382.81	30.32
L		
White	391.02	31.80
Red	424.22	31.80
Green	391.02	31.80
B		
White	356.58	38.01
Red	413.09	35.55
Green	362.85	33.52
Y		
A		
White	409.83	29.03
Red	366.86	29.03
Green	400.72	29.03
L		
White	393.23	29.03
Red	396.48	29.03
Green	402.70	30.32
B		
White	360.03	29.03
Red	381.51	29.03
Green	351.56	33.52

ANOVA test: group F 0.56 (DF 1) n.s.; VE (DF 2) F 0.65 n.s.; stimuli (DF 2) F 0.58, n.s.; group × stimuli (DF 2) F: 0.67 n.s.; group × VE (DF 2) F 0.06 n.s.; group × stimulus × VE (DF 4) F 0.52 n.s

### P3b amplitude

The MANOVA analysis showed a significant effect of group, stimulus type and for the interaction group × stimulus (MANOVA test: effect of group (DF 1) F (Roy radix) 2.009, DF 57 p 0.007; stimuli (DF 3) F 2.46 DF 57, p 0.001; groups × stimuli (DF 4) 1.94 DF 57, p 0.009). The effect of Virtual Environment was also significant as regard to P3b amplitude of target stimuli, but the interaction groups × VE was not significant though it approached the significance. (MANOVA group (DF 1) F (Roy Radix) = 2.46 DF 57 p 0.016; VE (DF 2) F 2.080 DF 57 p 0.036; groups × VR (DF 3) F 1.83 DF 57 p 0.068 n.s.)

**Fig. 5:**
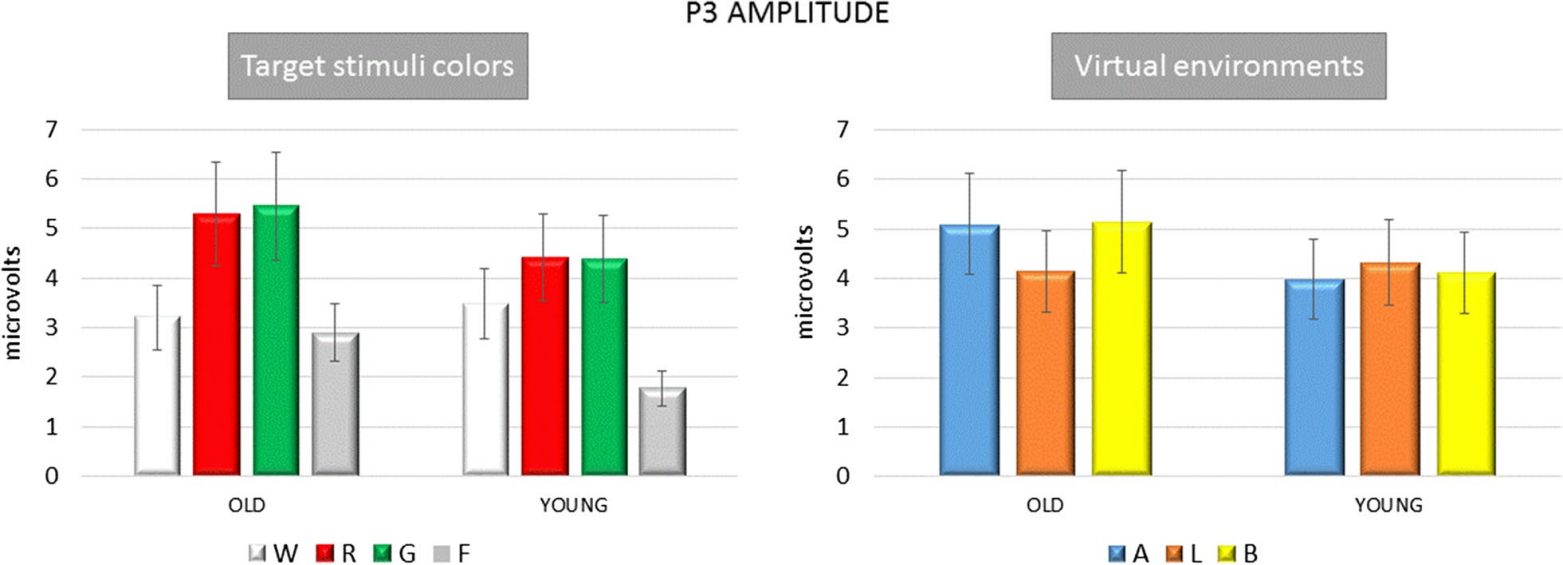
Mean and standard errors of P3b amplitude on Pz EEG channels in the Young and Old groups under different stimulation conditions. Virtual environments The P3b amplitude averaged across target stimuli is reported *A* Aisle; *L* living room; *B* bedroom. Target stimuli colors: The P3b amplitude obtained by target stimuli in *W* white, *R* red, and *G* green conditions are reported, as well as the amplitude of the positive response in the 300–600 ms time interval after *F*—frequent stimuli

**Fig. 6:**
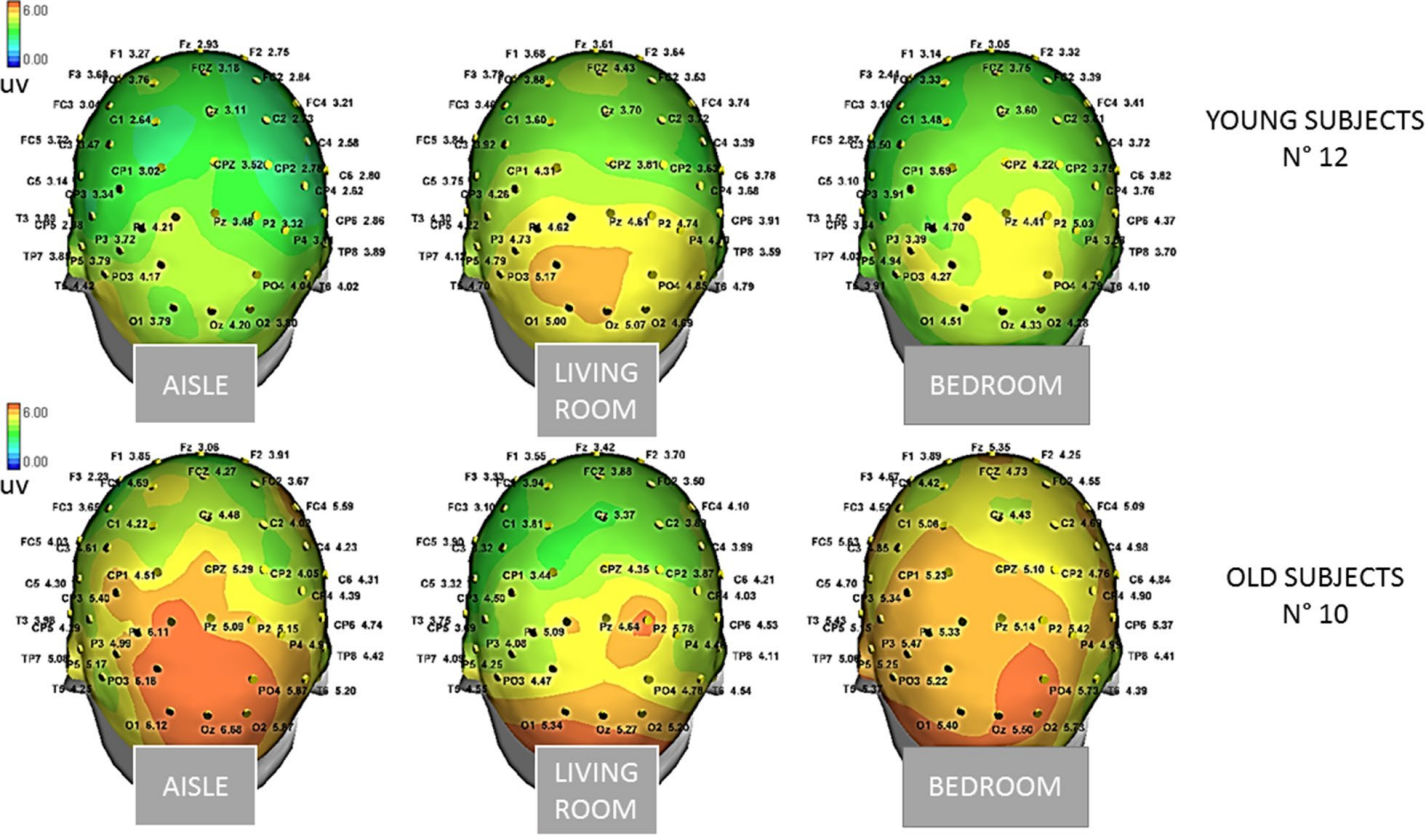
The grand average of P3b response amplitude by target stimuli in the different virtual environments are represented by a scalp map model provided by ASA software

In the Young group, the P3b amplitude did not appear clearly different among VE conditions, while in Old group the P3b appeared more diffused over the parietal and central derivations during the vision of target stimuli into the Aisle and Bedroom Virtual Environments (Figs. 5, 6). However, the Bonferroni test did not show significant differences among the different virtual context in any group.

**Fig. 7:**
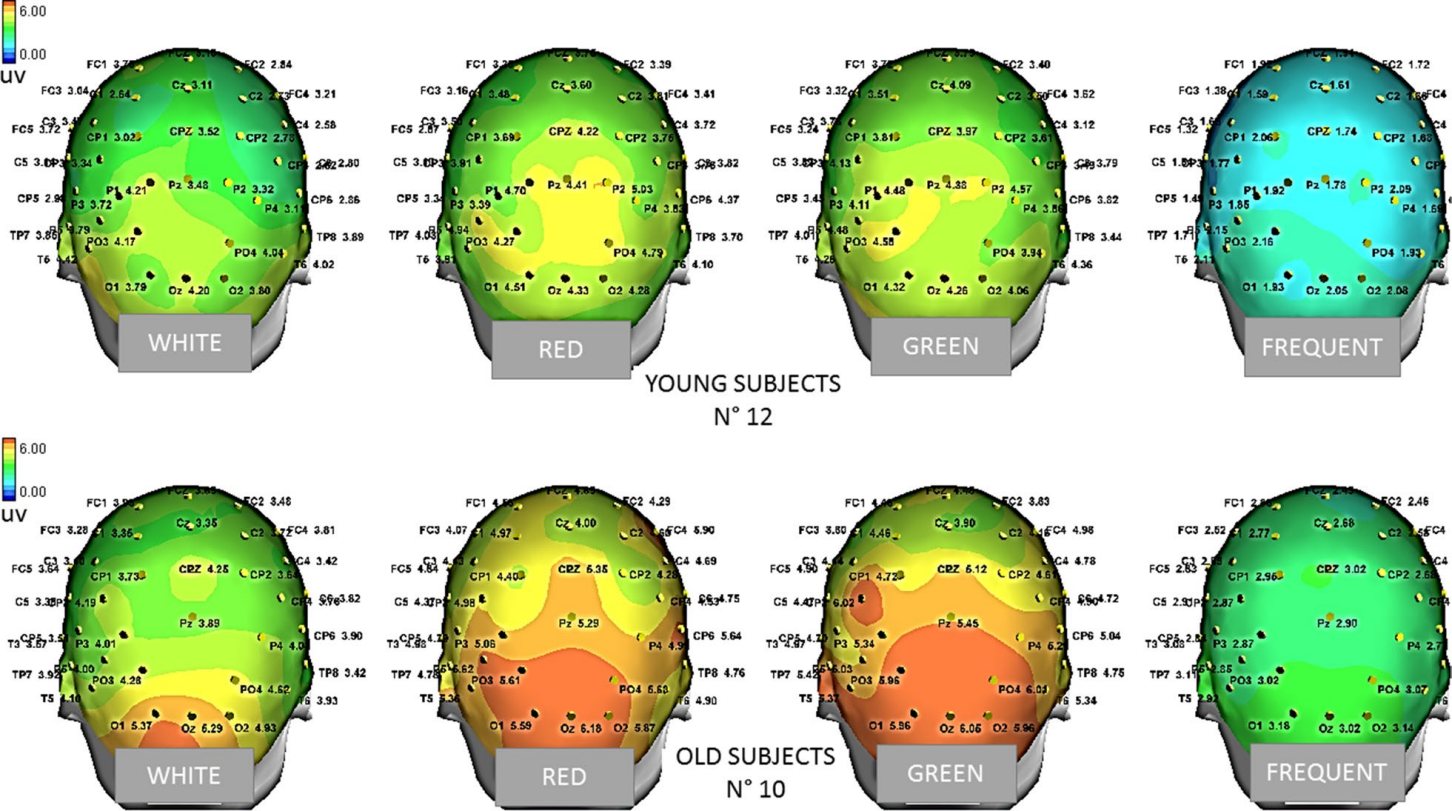
The grand average of the P3b response amplitude of the target and frequent stimuli are represented by a scalp map model provided by ASA software

**Fig. 8:**
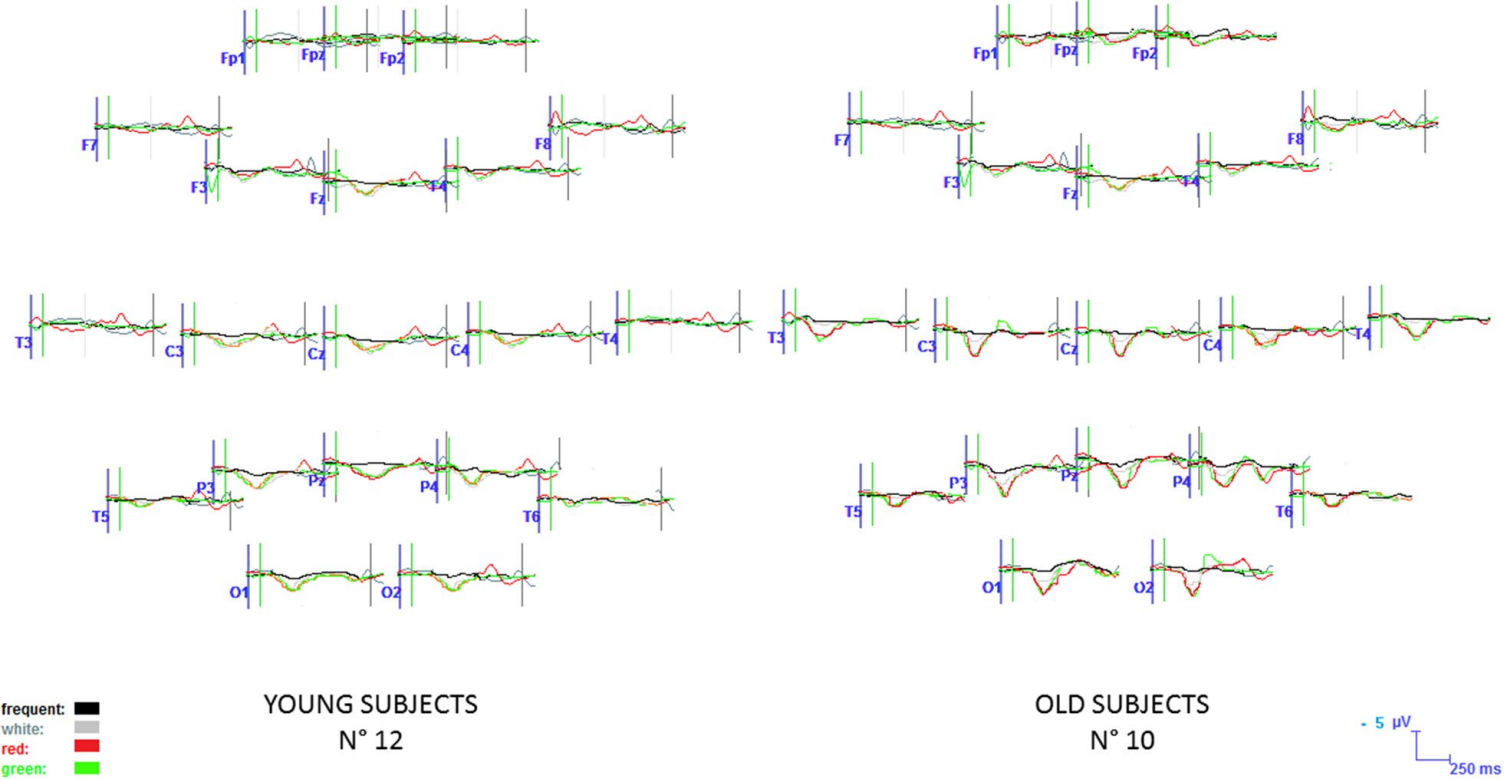
The grand average of the P3b response obtained by the target and frequent stimuli are represented on 20 representative channels in the two groups. In the old subjects, the response by the target stimulus in the White condition, represented in gray, is hardly distinguished by the response to frequent stimuli (represented in *black*), while the P3b by target stimuli in Red and Green conditions (represented with these *colors*) is clearly different from the response to the standard one. In the young subjects, the P3b by all the target stimuli is clearly recognizable from the response by the standard one

**Fig. 9:**
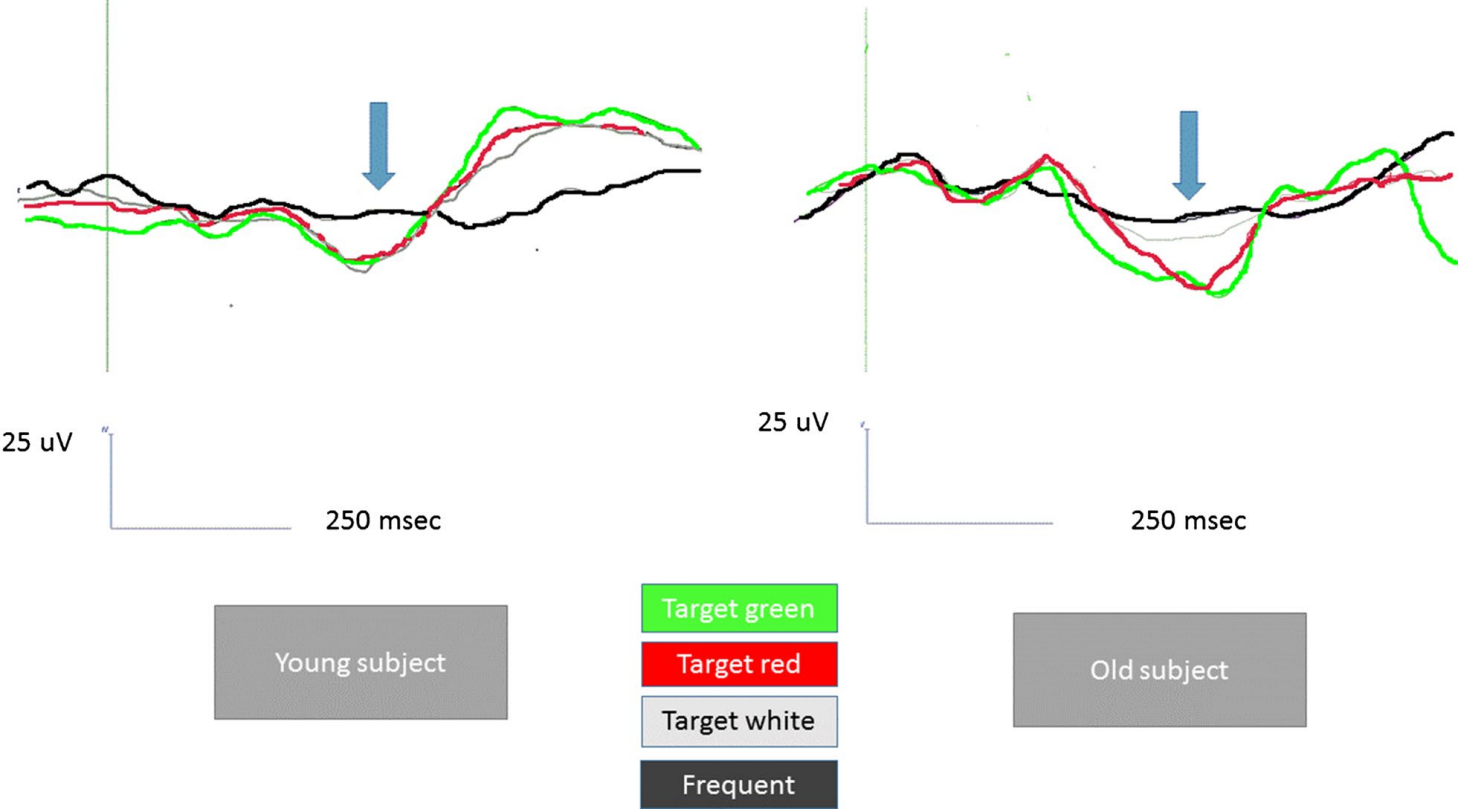
A detailed example of the P3b responses in 2 representative subjects (Pz channel). In the old subject, male, 75 years old, the response by the target stimulus in the White condition, is reduced in amplitude in respect to the response in red and green conditions (represented with these *colors*). In the young subject, male, 30 years old, the target P3b waves have a similar amplitude, independently from the color of the stimulus

**Fig. 10:**
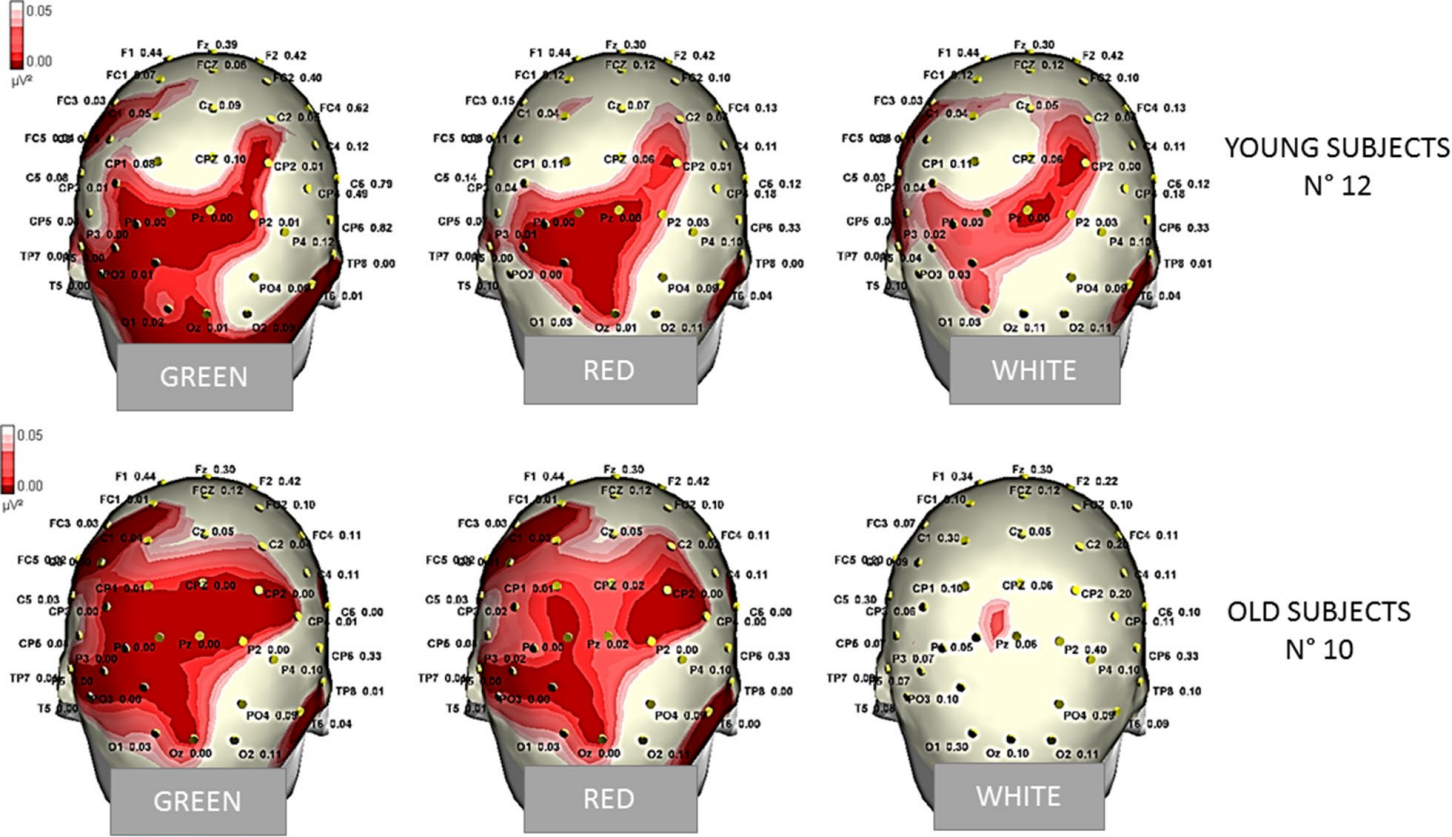
Statistical Probability Maps representing the results of the Bonferroni test comparing the P3b amplitude by the different target stimuli with the response obtained by standard stimuli in single groups. The red colors represent the significant p values

In the Young group, all the target stimuli determined a significant increase in P3b amplitude compared to the Frequent stimuli condition, whatever was the color of the target door (Figs. 5, 7, 8, 9). The Bonferroni test showed that the P3b was significantly increased on the parietal, occipital and central electrodes in W, R and G conditions, compared to F (Fig. 10).

The Bonferroni test showed also that in the elderly group the P3b obtained by the green and red colors were significantly different from the frequent stimulus, on the parietal, occipital, and central derivations, while the White stimulus did not evoke a significantly larger P3b with respect to frequent stimulus (Figs. 5, 7, 8, 9, 10).

In addition, the P3b by White door was significantly different from that obtained in the G and R conditions (W vs. G p < 0.05 on P3, O1, P7, T6, C7 derivations; W vs. R p < 0.01 on P3, O1, P7, T6, C7, CP3, <0.01 on Pz, Cp1, Cpz derivations).

## Discussion

The results of the present study may confirm in a preliminary way the reliability of a VR model to extract a P3b component and evaluate the possible age-related cognitive impact of simulated architectural context changes easily applicable to a real setting.

### Aim 1

The major difficulty of our model consisted in the synchronization of the appearance of the target stimulus in the visual field of a virtual walking to the EEG recording, to test the best position and color of target door to be reproduced in a real context. The response seemed to be coherent in terms of latency with a timely vision of stimuli and its recognition, according to a P3b potential (Polich 2007). The P3b features seemed also comparable with those reported in previous studies employing virtual reality paradigms by Brain Computer Interface models integrated with the online detection of evoked responses. (Bayliss and Ballard 2000; Bayliss 2003; Tarnanas et al. 2012; Chen et al. 2014, Rohani et al. 2014, Käthner et al. 2015) The usefulness of VR in cognitive rehabilitation was previously recognized (Bamdad et al. 2015), although the novelty of our stimulation method was the possibility to virtually reproduce a real architectural context to improve the habitual living environment by home automation technologies.

However, the virtual walking performed in the course of wayfinding created some movement artifacts and also subjective discomfort, especially in the elderly group, so the model could be further improved. In addition, at this stage, we did not evaluate aged people with cognitive impairment, which may further compromise the quality of recordings. The visual and automatic analysis detected the presence of a positive response in the expected time interval (Polich 2007), after different artifacts corrections, including the ICA, were applied off-line to discard all the stimulus-related EEG components linked with ocular or somatic movements. This off-line processing led to the exclusion of many cases, for the elimination of many EEG tracks useful for the next step analysis. In this preliminary work, we also used a 57 channels EEG recording to test the topographic reliability of the P3b and the possible modification under the employed experimental conditions, while few electrodes were applied in experimental settings based on cognitive evoked responses during VR tasks (Bayliss and Ballard 2000; Bayliss 2003; Rohani et al. 2014). The topographical distribution of visual P3b we obtained indicated a prevalent representation over the posterior parietal electrodes, with the involvement of occipital derivation and diffusion over the central regions, which is coherent with the P3b maps we previously obtained by a standard visual paradigm (de Tommaso et al. 2008). Previous studies, dealing with the topographical representation of visual P3b obtained in a BCI stimulation frame by both EEG and MEG, indicated the same regions as discriminant in regard to the positive cognitive response (Bianchi et al. 2010), confirming the reliability of our VR paradigm for the extraction of this potential. Our statistical maps showed a significant amplitude modification in the expected time range related to target stimuli versus non-target on the same regions, with a prevalent involvement of the left hemisphere in both groups, which is coherent with an asymmetric activation of the 2 hemispheres during the visual P3b task (Ji et al. 1999).

### Aim 2

We did not observe significant differences in regard to the P3b latency between the young and old groups, which is in contrast with the progressive latency increase generally described in old people (Mueller et al. 2008). Our task was quite easy, as the bathroom was designed in order to be simply recognized during a virtual walking while age-related differences in P3 features are increased with the level of task difficulty (Speer and Soldan 2015). Our old subjects were also intellectively normal and with a medium–high level of education, and in previous studies, the speed of cognitive processing subtending the P3b was not reduced in high performers aged subjects, who can enhance their cognitive abilities employing compensatory neural activities (Riis et al. 2008). In addition, the most of the studies comparing the P3b across different ages were performed by standard visual or auditory stimulation paradigms, while the VR model may facilitate the cognitive task and increase the recognition of target stimuli. Latencies were also not influenced by different virtual environments and target colors, confirming that the bathroom door was a simple and familiar object whatever was its color or the architectural context where it was included.

Accordingly, we did not find a P3b amplitude decrease as the effect of group, but a different modulation between groups was present as effect of virtual conditions. In particular, we observed that in the old group the P3b amplitude was higher over the parietal and central zones when the target door was included into a more expectable and familiar context, like the corridor or the bedroom. This phenomenon was absent in younger subjects, though the interaction between group and virtual environment on P3b amplitude only approached the statistical significance. Despite the strength of this different behavior was not so relevant to determine a significant result of Bonferroni test, the increased diffusion of the P3b over the parietal and central scalp locations, obtained when the target door was inserted into an ordinary setting, may confirm that a familiar context may facilitate a cognitive task in elderly (Rémy et al. 2013). The anterior shift of the P3b, usually observed in old age for compensatory cortical mechanisms useful to successfully perform the cognitive task (Pfefferbaum et al. 1984; Fabiani and Friedman 1995), was facilitated in our aged subjects when the target door was in a more familiar context. The reasons for the lack of significance may be different; firstly the small subjects series, furtherly the employment of a reliable and easily recognizable house ambient, able to reduce the impact of the unusual target door location, and lastly the effect of target colors, which contributed to enhancing the P3b amplitude.

In fact, the colors of the target doors seemed an element which differentiated old from young groups. The older subjects were unable to respond with a clear P3b wave when the target stimulus was simply illuminated with a white spotlight while their cognitive response was clear and large when the red and green lights indicated the target door.

An age-related decline in the ability to differentiate targeted from standard stimuli was previously observed, and even normal aging may be characterized by a failure to recruit specialized neural modules and generate differentiated neural responses to various classes of stimuli responses (Mott et al. 2014). So far, old people may have the need to be facilitated in the wayfinding of a very simple target place to produce a P3b response comparable to young subjects. Visual search processing is compromised with advanced age (Lorenzo-López, et al. 2008), so stronger color and luminance contrasts are needed to reinforce the recognition, even in the case of a very familiar and easy target place.

Our old subjects were cognitively normal and not affected by visual problems, which determined a normal P3b latency and amplitude. The modulation of P3b amplitude, obtained by color and luminance change of target place, suggests that cortical resources, able to compensate the age-related progressive loss of cognitive performance, need to be facilitated even in normal elderly.

## Conclusions

The results of our study suggest that ERP obtained by virtual reality is a useful and reliable tool to test cognitive functions and age-related cognitive changes, despite some technical problems have to be resolved, to make the tasks more comfortable and avoid artifacts.

The advantage of such device may be the simulation of real architectural contexts and possible changes aiming to facilitate orientation and wayfinding. Our elderly subjects demonstrated to be influenced by the colors and light changes, easily performable in a real ambient also by remote home automation control, which can hypothetically create the best environmental conditions in a dynamic and feasible model adaptable to individual cognitive dysfunctions.

These results need to be validated in cohorts of cognitively impaired elderly patients, in order to find a possible method to improve ambient assisted living in housing and health facilities.
